# The cell biology of *Tobacco mosaic virus* replication and movement

**DOI:** 10.3389/fpls.2013.00012

**Published:** 2013-02-11

**Authors:** Chengke Liu, Richard S. Nelson

**Affiliations:** Plant Biology Division, The Samuel Roberts Noble Foundation, Inc.Ardmore, OK, USA

**Keywords:** membrane transport, microfilaments, microtubules, plant virus, vesicle trafficking, tobamovirus

## Abstract

Successful systemic infection of a plant by *Tobacco mosaic virus* (TMV) requires three processes that repeat over time: initial establishment and accumulation in invaded cells, intercellular movement, and systemic transport. Accumulation and intercellular movement of TMV necessarily involves intracellular transport by complexes containing virus and host proteins and virus RNA during a dynamic process that can be visualized. Multiple membranes appear to assist TMV accumulation, while membranes, microfilaments and microtubules appear to assist TMV movement. Here we review cell biological studies that describe TMV-membrane, -cytoskeleton, and -other host protein interactions which influence virus accumulation and movement in leaves and callus tissue. The importance of understanding the developmental phase of the infection in relationship to the observed virus-membrane or -host protein interaction is emphasized. Utilizing the latest observations of TMV-membrane and -host protein interactions within our evolving understanding of the infection ontogeny, a model for TMV accumulation and intracellular spread in a cell biological context is provided.

## INTRODUCTION

Viruses, as obligate organisms, utilize host factors to accumulate and spread in their host. A successful infection by a plant virus includes entry and accumulation in the first cell, movement into neighboring uninfected cells, and systemic infection through the plant vascular tissue (Boevink and Oparka, 2005; Epel, 2009; Harries and Ding, 2011; Niehl and Heinlein, 2011; Schoelz et al., 2011; Tilsner et al., 2011). Plant viruses have varying strategies for infecting hosts which reflect their use of existing functionally redundant host developmental pathways. Therefore an understanding of virus infection processes also offers insight into normal host physiological processes. *Tobacco mosaic virus* (TMV) encodes four known functional proteins: the 126 and 183 kDa replication-associated proteins, the movement protein (MP), and the structural capsid or coat protein (CP). In order to have a successful infection, these four multifunctional proteins cooperate with many host components. The host membrane and cytoskeleton are sub-cellular structures important for TMV infection. TMV-induced granules or inclusion bodies that contain membranes also contain host proteins. In this review, we discuss the changing roles of host membranes, cytoskeleton, and inclusion body-associated proteins as infection progresses. Findings reported in the literature are first presented in the section(s) where the effect on virus physiology was observed rather than where it may additionally influence this activity. For example, the influence of synaptotagmin on TMV physiology (Lewis and Lazarowitz, 2010) was reported as an inhibition of intercellular spread of the TMV MP, although it likely influences the intracellular transport of this protein. This was done to clearly indicate what is in the published literature rather than what a reader may interpret the results to indicate. In some instances, however, the presumed influence of the observed outcome on the mechanism of virus movement is noted. As pertinent, findings from other tobamoviruses are mentioned to indicate the generality or specificity of a conclusion for the genus.

## INITIAL INFECTION

**FIGURE 1:**
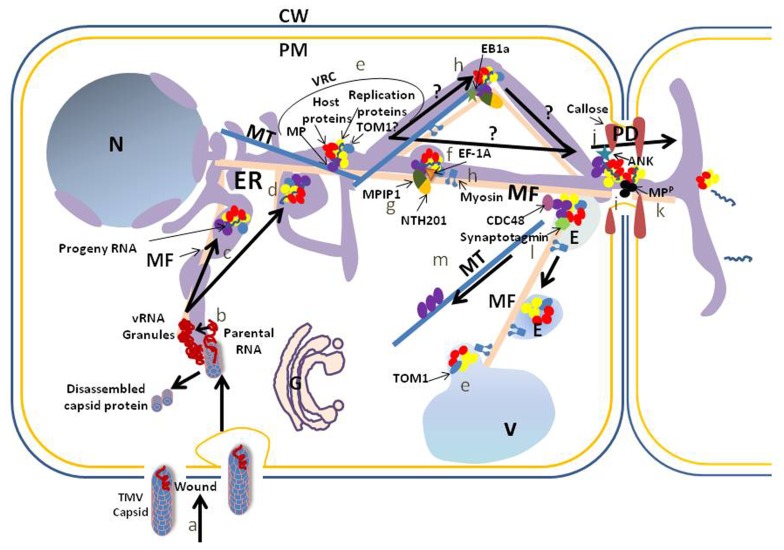
**Schematic of a proposed accumulation and movement pathway for TMV within cells.** To simplify the model we do not address the possibility that the MP or any other viral protein moves within the cell, with or without viral RNA, independently of the virus replication complex. Also, we do not address the possibility that host proteins involved in virus accumulation and movement traffic independently from the virus complexes to support these activities. TMV capsid enters through an opening within the cell wall (CW) and plasma membrane (PM) or through pinocytosis after wounding (a). TMV RNA is released from the capsid at the site of viral RNA (vRNA) granule formation (b). The granules are associated with the endoplasmic reticulum (ER), which may serve as the replication site on transport of the vRNA to cortical vertices or perinuclear regions of the ER. Transport to these locations requires microfilaments (MF) (c). Other membranes such as the vacuolar (V) membrane may serve as a scaffold for virus replication, but this requires further analysis. A virus replication complex (VRC) is formed in the cortical vertices or perinuclear region of the ER (d). VRCs contain vRNA, movement protein (MP), replication proteins and host proteins. TOM1, a membrane protein, interacts with replication proteins and serves as an anchor between the replication proteins and a host membrane, which may be ER (TOM1?), vacuole (TOM1) or another membrane (e). For TMV intercellular movement, VRCs move from sites of replication to plasmodesmata (PD). Elongation factor 1A (EF-1A) interacts with vRNA, replication proteins, MFs and microtubules (MTs) and influences TMV movement. It is unclear if this influence is on sustained movement associated with clearance of virus components within the cell, or with initial movement: we have placed it with initial movement and with the MF (f). An interaction between two host proteins, a class II KNOTTED 1-like protein (NTH201) and a DnaJ-like protein (MPIP1), and the TMV MP also may aid transport of virus to the PD (g), although again it is unclear if this interaction aids initial or sustained movement. Movement of the VRC to the PD requires membrane, and may be influenced by actomyosin (MF and myosin) and MT (h). The influence of the MT end-binding protein (EB1a) on virus movement is placed during transport to the PD (h). MP microfilament severing activity at the PD is proposed to eliminate F-actin-like structures at the PD to increase the PD size exclusion limit SEL (i). In addition, interaction between the MP and the ankyrin repeat containing protein (ANK) is correlated with an increase of the PD SEL through a decrease in callose at the PD neck (j). vRNA transports through PD within a VRC or simply with vRNA and MP, the latter being phosphorylated (MP^P^) in the PD to release the vRNA for translation in the next cell (k; Karpova et al., 1997; Lee et al., 2005). After vRNA transfer to the neighboring cell, VRC remnants associate with endosomes (E; possibly pre-vacuolar vesicles) for transport to vacuoles, potentially through interaction of the vesicle fusing protein, synaptotagmin, with the MP (l). Transport is proposed to involve the actomyosin network. Likely prior to this transport, CELL-DIVISION CYCLE protein48 (CDC48) extracts the MP from the ER-associated VRC for attachment to the MT and later degradation (m). N, nucleus.

*Tobacco mosaic virus* enters plant cells only through mechanical wounds which either transiently open the plasma membrane or allow pinocytosis (Palukaitis and Zaitlin, 1986; Shaw, 1999; **Figure 1**). TMV begins to disassemble within 3 min after entry and disassembly of CP from the capsid is associated with translation of viral RNA (vRNA; Wu et al., 1994; reviewed in Shaw, 1999). TMV vRNA labeled with cyanine 3-UTP forms small granules in cytoplasm less than 5 min after entering the cell (Christensen et al., 2009). The vRNA-containing granules form where CP and vRNA co-localize as well as in the absence of CP, suggesting that although CP was not needed for granule formation the disassembly of TMV capsids occurred at the site of granule formation. Removal or mutation of *cis* and *trans* elements necessary for virus replication (i.e., the vRNA 3′ untranslated region and replicase) did not prevent granule formation, although they were smaller and less stable. The granules were shown to associate with fluorescently labeled ER. The 5′ methylguanosine cap on the vRNA was necessary to anchor vRNA to the ER/actin complex: absence of the cap leading to vRNA degradation and no granules (Christensen et al., 2009). Considering that uncoating of vRNA may make it accessible to the silencing surveillance system (reviewed in Niehl and Heinlein, 2011), it will be important to determine to which host factors viral proteins are attached during granule formation and transport to cortical and perinuclear replication sites. Identifying host factors in the granules will be difficult due to their presumed low quantities, but will be necessary to understand the steps prior to virus replication.

## REPLICATION

*Tobacco mosaic virus* and the very closely related *Tomato mosaic virus* (ToMV) use their parental genomes to synthesize complementary negative strands which serve as templates for the synthesis of progeny full-length positive strands and subgenomic mRNAs containing MP and CP open reading frames (ORFs; Ishikawa and Okada, 2004; Ishibashi et al., 2010). Although the 183 kDa protein encoded by the 5′ ORF of these viruses can replicate the genome, the 126 kDa protein, produced by termination of translation at an amber stop codon within the 183 kDa protein ORF, is necessary for maximum progeny RNA production (Ishikawa et al., 1986; Ishikawa et al., 1991; Lewandowski and Dawson, 2000). The 126 and 183 kDa proteins contain methyltransferase and helicase domains, while the 183 kDa protein alone contains the C-terminal domain encoding an RNA-dependent RNA polymerase. The 126 and 183 kDa proteins together will be referred to as the replication proteins in this review. TMV/ToMV replication is believed to occur in a membrane-associated complex containing the replication proteins, MP, vRNA, and host proteins (Hagiwara et al., 2003; Heinlein et al., 1998; Más and Beachy, 1999; reviewed in Ishibashi et al., 2010; Laliberté and Sanfaçon, 2010; de Castro et al., 2012; **Figure 1**). The ER was implicated as a site for virus replication complex (VRC) formation through co-localization of an ER marker, BiP, with MP-GFP and in turn, MP-GFP co-localization with replication proteins during immunofluorescence studies (Heinlein et al., 1998). Reichel and Beachy (1998), using transgenic plants expressing an ER-targeted GFP, determined that the ER formed large cortical aggregates at reticulate vertices and fewer membrane tubules during TMV accumulation, but returned to a normal structure after replication ended. However, later studies with ToMV using both fluorescence microscopy and biochemical fractionation methods determined that the replication proteins and replicase activity were associated predominantly with the vacuolar membrane, although they also showed some localization and activity with other less defined membrane fractions which included the ER (Hagiwara et al., 2003). Interestingly, ToMV can replicate in cells that are vacuole-diminished (Nishikiori et al., 2006). This finding supports the notion that although the tonoplast may function to support ToMV/TMV accumulation, other membranes such as the ER are important for this activity. Clearly, additional work is necessary to determine which membranes are essential for tobamovirus accumulation (**Figure 1**).

Regardless of the membrane used for tobamovirus accumulation, it is clear that a characteristic VRC is not uniformly induced by tobamoviruses. TMV, ToMV, and *Tobacco mild green mosaic virus* (TMGMV), form different subcellular structures containing replication proteins late in the infection cycle: TMV forming X-bodies and ToMV and TMGMV forming virus bundles when analyzed through immunocytochemical EM studies (Das and Hari, 1992). For a fourth tobamovirus, *Turnip vein clearing virus* (TVCV), X-bodies are rare (Resconich, 1961). Early fluorescence localization studies determined that for strains of TMV the size of the VRCs varied and was positively correlated with the level of disease observed (Liu et al., 2005, 2006). Recently, however, it was determined that silencing expression of the gamma subunit of ATP synthase, a nuclear-encoded chloroplast protein, resulted in smaller but more numerous VRCs and severe disease symptoms (Bhat et al., 2012). Thus, the size of the VRC is not a perfect indicator of disease intensity and the number of VRCs may influence this phenotype.

The form of inclusions induced by tobamoviruses is correlated with differences in the replication protein sequences (Liu et al., 2005; Harries et al., 2009). While ectopically expressed 126 kDa protein from TMV fused with GFP forms intracellular inclusions, the 125 kDa protein homolog from TVCV does not form inclusions (Harries et al., 2009). Interestingly, the intracellular inclusions formed by the TMV 126 kDa protein co-localized with microfilaments, as observed for the TMV VRC (Liu et al., 2005). Domain(s) within the 126 kDa protein necessary for inclusion body formation are not identified, however, it is known that the helicase domain when expressed alone is able to form octomers *in vitro* (Goregaoker and Culver, 2003) and thus may be a domain important for this activity. In addition, a 126 kDa protein-GFP construct expressing only the N terminal 781 amino acids of the 126 kDa protein associated with the ER and formed inclusions (dos Reis Figueira et al., 2002). The 781 amino acid protein includes the methyltransferase and non-conserved bridge domain that previously was determined to influence the RNA silencing suppression function of this protein. There is unpublished data indicating that the methyltransferase domain alone can form inclusions (Knapp et al., 2007). More work is needed to further identify the domains responsible for inclusion formation and the relevance of inclusion formation to tobamovirus physiology.

Ectopically expressed TMV MP fused with fluorescent reporter proteins also can form cytoplasmic inclusion bodies (Heinlein et al., 1998; Reichel and Beachy, 1998; Sambade et al., 2008). These small inclusions are similar to those visualized in the cortical periphery during infection with tobamoviruses expressing an MP-GFP fusion (Padgett et al., 1996; Heinlein et al., 1998; Reichel and Beachy, 1998; Boyko et al., 2007). The cortical MP-GFP inclusions that appear during virus infection likely represent the inclusions containing replication proteins and MP observed by Szécsi et al. (1999), but this requires confirmation. Inclusions containing MP-GFP associate with microtubules both early and late in the infection cycle (Heinlein et al., 1998; Boyko et al., 2007; Sambade et al., 2008). Studies with cellular markers and an MP-mRFP construct containing a downstream non-viral stemloop-forming RNA aptamer that can be fluorescently labeled determined that MP-mRFP associates with ER and its own RNA (Sambade et al., 2008). In this regard, it will be important to determine if multiple types of inclusions are formed independently during infection or are always part of a continuum, with progeny inclusions appearing from parental inclusions.

The host proteins within VRCs or inclusions that contain them are not fully identified. Late in infection, TMV-induced X-bodies have been shown to contain the microtubule component, β-tubulin, through immunocytochemical EM studies (X. S. Ding and R. S. Nelson, personal communication). The function of this protein in X-bodies is unknown, but perhaps it could be to aid the degradation of body components analogous to the suggested function of microtubules during TMV MP turnover (Kragler et al., 2003). The host translation factor, elongation factor 1A (EF-1A), is present in the membrane-associated fraction where viral replicase activity was observed (Osman and Buck, 1996; Watanabe et al., 1999) and in X-bodies produced by TMV (Ding et al., 1998). It also interacts with the 3′-UTR of genomic vRNA and with the methyltransferase domain of the replication proteins (Zeenko et al., 2002; Yamaji et al., 2006). EF-1A has additional activities beyond supporting translation including forming complexes with tubulin and actin, the actin interaction possibly linking the cytoskeleton to protein synthesis, and ubiquitin-mediated degradation (Durso and Cyr, 1994; Gonen et al., 1994; Kim and Coulombe, 2010). The function of EF-1A during TMV accumulation was hypothesized to aid minus strand synthesis (Yamaji et al., 2006). However, down-regulation of EF-1A through virus-induced gene silencing resulted in the reduced size of green fluorescent lesions induced by TMV-expressing GFP, but no reduction in lesion numbers or translation activity in the silenced leaves (Yamaji et al., 2010). This result suggests that the function of EF-1A is not for translation or virus accumulation, but for virus movement that may, in some manner, be linked to the cytoskeleton (**Figure 1**).

Tobamovirus multiplication 1 (TOM1) is a predicted multi-pass transmembrane protein required for tobamovirus accumulation (Ishikawa et al., 1993; Yamanaka et al., 2000). Surprisingly, over-expression of TOM1 decreases ToMV multiplication (Hagiwara-Komoda et al., 2008). This observation is associated with the finding that accumulation of the ToMV replication proteins in membrane-free (soluble) fractions was lower for plants over-expressing TOM1 compared with those not over-expressing this protein (Hagiwara-Komoda et al., 2008). This result indicates that the level of the soluble form or the ratio of soluble and membrane-bound forms of the replication proteins is critical for normal virus accumulation. It was hypothesized that the soluble form is important for RNA silencing suppressor activity and it was shown that the loss of suppressor activity is correlated with diminished accumulation of the virus (Kubota et al., 2003; Hagiwara-Komoda et al., 2008). TOM1 interacts with the helicase domain of the 130 kDa protein from the related tobamovirus, *Tobacco mosaic virus-Cg* (crucifer-infecting virus), in a yeast two-hybrid screen (Yamanaka et al., 2000). This interaction was shown to be with the helicase core region based on predictions from the crystal structure of the helicase domain (Nishikiori et al., 2012). The replication proteins from ToMV and TOM1 share similar subcellular fractionation pattern in extracts from infected BY-2 cells, residing mostly in the tonoplast-containing fractions, but also in fractions with other membranes, including the ER (Hagiwara et al., 2003). It is hypothesized that TOM1 forms a link between the host membrane in which it resides and the tobamovirus replication proteins (**Figure 1**). This interaction is likely important for VRC formation, but the co-localization of TOM1 and tobamovirus replication proteins in live cells has not been reported.

## INTRACELLULAR MOVEMENT

For TMV to establish a systemic infection, the virus or its components must move within a cell to establish an infection site, multiply and finally position for movement to the next cell. The granules of vRNA that form on initial infection, the VRCs that form during infection and the 126 kDa protein- and MP/vRNA-containing inclusions observed during ectopic expression all move within the cell (e.g., Kawakami et al., 2004; Liu et al., 2005; Sambade et al., 2008; Christensen et al., 2009). These complexes may change their form and constituents with time.

During initial infection, granules containing vRNA anchor to cortical ER and move to cortical ER vertices and the perinuclear ER where virus replication and translation occurs (Reichel and Beachy, 1998; Christensen et al., 2009; **Figure 1**). Indeed, vRNA has been visualized in the perinuclear bodies by bimolecular fluorescence complementation using a modified sequence-specific RNA-binding protein, Pumilio1, or by classical *in situ* RNA labeling (Tilsner et al., 2008). The vRNA must contain a sequence that targets the ER membrane directly or through a protein that targets the ER. Why the vRNA moves to particular cortical ER vertices or the perinuclear ER for replication is unknown. However, considering that most of the ribosome-containing, or rough, ER is present in the perinuclear region (Carrasco and Meyer, 2011) it is likely that this location, or a cortical ER vertex also containing ribosomes, is best suited for virus protein synthesis. Neither cytochalasin D nor latrunculin B (LatB) treatment, both microfilament antagonists, affect granule formation suggesting that microfilaments are not involved in this initial activity (Christensen et al., 2009). However, disruption of microfilaments results in granules hovering in the cortical ER, suggesting microfilaments help transport the granules in the cell. In contrast, depolymerizing microtubules does not stop vRNA granule movement along the tubular cortical ER (Christensen et al., 2009).

Membranes may be involved in intracellular trafficking of TMV components and the virus itself, as a VRC or vRNP, during virus accumulation and later spread. TMV replication occurs in association with ER and other membranes and both the MP and the replication proteins associate intrinsically or through a protein linker with membranes (Brill et al., 2000; Hagiwara et al., 2003; Fujiki et al., 2006). Interestingly, however, interruption of ER to Golgi secretory transport, mediated by the host coat complex II (COP-II) with brefeldin A (BFA) or through over-expression of a dominant-negative GTPase, Sar1p, did not alter targeting of ectopically expressed, fluorescently labeled MP to plasmodesmata (PD; Boutant et al., 2009; Genovés et al., 2010). This result, as well as BFA studies with infectious virus (see below), indicates that the COP-II-mediated transport system is not utilized by TMV MP or TMV to target viral products to the cell periphery. The actual pathway used by the virus, however, likely includes ER since that membrane is present in early- and late-formed virus inclusions (by fluorescence microscopy and EM), with early forming inclusions paired at the cell wall (e.g., Esau and Cronshaw, 1967; Heinlein et al., 1998; Szécsi et al., 1999; see below).

Much information is available on the movement of inclusion bodies containing the viral replication proteins. Several laboratories pursued EM-based immunocytolocalization studies of TMV infections with antibody against the replication proteins (Hills et al., 1987; Saito et al., 1987). They noted that the structure of the inclusions likely changed during development, going from smoothly granular to containing electron-dense rope-like structures, composed at least partly of 126 kDa protein, in a ribosome-rich matrix. Saito et al. (1987) referred to the former as viroplasms and the latter as X-bodies. Szécsi et al. (1999) showed through light and EM immunocytolocalization studies that TMV-induced inclusion bodies with viroplasm and X-body characteristics changed position and content as infections within cells developed. Near the infection front the inclusion bodies were paired on either side of the cell wall and contained both the replication proteins and MP, while four to six cells back from the front the bodies were not paired, had moved away from the cell wall and only contained the replication proteins. Through fluorescence microscopy of cells near the infection front, Tilsner et al. (2008) observed vRNA in small cortical bodies in the peripheral cytoplasm. They suggested these cortical bodies may represent the inclusions at the cell periphery observed by Szécsi et al. (1999). Motionless small fluorescent bodies in the cell were detected at 12 h post-inoculation and these bodies were moving by 14 h post-inoculation when tracking TMV expressing an MP-GFP fusion: a period when both MP and the replication proteins would co-localize (Kawakami et al., 2004). Movement of the fluorescent inclusions early in infection, when both replication proteins and MP would be present, was aligned with microfilaments, and through pharmacological and gene-silencing studies, the inclusions (also referred to as VRCs) were shown to require these intact microfilaments for their intracellular movement (Kawakami et al., 2004; Liu et al., 2005). The degradation of the microfilaments was not sufficient to decrease virus accumulation to levels that would prevent virus movement or VRC formation and thus the influence of microfilaments on TMV intracellular movement was not confounded by a significant inhibition of virus replication (Liu et al., 2005). Treatment with a general myosin motor inhibitor, 2,3-butanedione monoxime (BDM), impaired the intracellular movement of VRCs (Kawakami et al., 2004).

Boyko et al. (2007), using a chimeric TMV expressing the MP from the Ob tobamovirus (Padgett et al., 1996), determined that MP-GFP inclusions at the infection front likely representing VRCs were associated with and typically trafficked along cortical microtubules for short distances in a stop-and-go manner (microtubules labeled with a microtubule-associated protein fused with GFP). Later studies obtained similar findings using the native MP of TMV fused with mRFP (Sambade et al., 2008). It was hypothesized that microtubules serve to anchor and then, through polymerization, release the VRC for movement (Sambade and Heinlein, 2009; reviewed in Peña and Heinlein, 2012). A second hypothesis, to be discussed further in the section on TMV intercellular movement, was that microtubule polymerization pushes the VRC along the ER (Peña and Heinlein, 2012). Other research, however, found that disruption of microtubules through pharmacological treatment with aminoprophos-methyl, colchicine, or oryzalin, or by silencing α-tubulin, had no significant effect on the transport of the MP-GFP within cells or to the PD area during infection (Gillespie et al., 2002; Kawakami et al., 2004; Wright et al., 2007). Regarding microfilaments, early studies indicated an association of TMV MP with rhodamine-conjugated phalloidin microfilaments after probing cells with polyclonal antibody against the MP and fluorescein-conjugated secondary antibody (McLean et al., 1995). Wright et al. (2007) utilized fluorescence recovery after photobleaching (FRAP) to observe MP-GFP movement and found that microfilament antagonists, LatB and cytochalasin B, inhibited MP targeting to the PD. However, a later study determined that the MP-GFP expressed during virus infection was not observed to co-align with fluorescently labeled microfilaments (Hofmann et al., 2009). Lastly, in studying membrane-mediated transport of the MP, inhibition of ER–Golgi membrane trafficking with BFA at low concentration (10 μg/ml) did not inhibit MP targeting (Tagami and Watanabe, 2007; Wright et al., 2007), but did influence the structure of the MP-GFP inclusion bodies (Tagami and Watanabe, 2007). At high concentration of BFA (100 μg/ml), which disrupts cortical ER structure, there was a significant effect on MP targeting to the PD (Wright et al., 2007). Thus, during virus infection the trafficking of the MP within the cell likely requires intact ER and may require microfilaments and microtubules (**Figure 1**), although evidence against a direct action for microfilaments exists and microtubules may not be in an intact form or the required microtubule array is unusual in that it is impervious to certain pharmacological agents (Seemanpillai et al., 2006).

Although the TMV VRC moves within cells, the replication proteins and the MP, independent of one another and in the absence of vRNA, also can transport within the cell. Their independent transport may have physiological relevance. Expression of a fusion of the TMV 126 kDa protein with GFP yields an intracellular inclusion body that, like the VRC, co-aligns with and traffics along microfilaments (Liu et al., 2005). As observed for the VRC, disruption of microfilaments ends intracellular transport of the 126 kDa protein-GFP inclusion body. Furthermore, normal intracellular movement of the 126 kDa-GFP inclusion body, like TMV sustained intercellular movement, requires myosin XI-2 (see section on “INTERCELLULAR MOVEMENT” for discussion of myosin influence on TMV physiology; Harries et al., 2009; C. Liu, and R. S. Nelson, personal communication). Whether the 126 kDa protein directly interacts with microfilaments or myosin XI-2 requires further study. If there is no direct interaction between these proteins, trafficking of virus proteins may be through interaction of myosin XI-2 with host components associated with a virus-host protein complex or through the creation of a bulk flow network of cytoplasmic constituents (i.e., cytoplasmic streaming). Considering that the viral replication proteins may transport independently of the MP or other viral components to complete their functions, additional studies of their ectopic transport in relationship to their transport during virus infection are needed. The difficulty in pursuing studies on the viral replication proteins is that no fusion between the replication proteins and a fluorescent marker has been developed that yields a viable virus. This needs to be addressed for further progress to occur.

Transient expression of the MP fused with fluorescent markers results in the formation of inclusions that associate with RNA and associate with and traffic along the ER, perhaps interacting with a microtubule scaffold necessary for movement (Sambade and Heinlein, 2009). Both microfilament and microtubule antagonists inhibited intracellular transport of the MP-fluorescent marker fusion, the results from the latter treatment (using aminoprophos-methyl) being a different finding from many during virus infection where MP-GFP fusion movement was not impeded by microtubule antagonists. To complicate this situation further, additional research determined that neither oryzalin or aminoprophos-methyl, both antagonists of microtubules, nor LatB, a microfilament antagonist, inhibited the formation of MP-GFP inclusions or their localization to the cell periphery or PD in cultured cells or leaves (Prokhnevsky et al., 2005; Boutant et al., 2009). These apparently conflicting results highlight the difficulty interpreting findings from pharmacological studies. Clearly, additional work is required to determine, in real time and through methods using non-pharmacological techniques, the influence of the cytoskeleton on transiently expressed MP intracellular trafficking. Assuming MP trafficking independent of the VRC has physiological relevance, findings from these additional studies would provide further insight into the mechanism of TMV intracellular movement. Some work investigating the influence of MP transport in the absence of pharmacological treatment has been published. For example, Kotlizky et al. (2001) determined that an ectopically expressed MP mutant-GFP fusion, MP^NT-1^-GFP, which inhibits TMV spread when expressed in transgenic plants, was able to associate with microtubules but did not target PD or move between cells. This suggests that the microtubule binding domain resides in a different location from the region important for PD localization and supports the pharmacological studies indicating that MP PD localization and initial virus spread requires more than microtubule association by the MP.

## INTERCELLULAR MOVEMENT

As for intracellular movement, our understanding of TMV intercellular movement is fragmented. It is certain, however, that TMV utilizes PD to move between cells. PD are bounded by the plasma membrane and contain a cytoplasmic sleeve between this membrane and intact ER, the ER referred to as the desmotubule in this tissue (Lucas et al., 2009; Benitez-Alfonso et al., 2010; White and Barton, 2011; **Figure 1**). Callose is present in the neck region of the PD (Northcote et al., 1989; see Benitez-Alfonso et al., 2010). Actin and myosin are among multiple protein components in the cytoplasmic sleeve (Fernandez-Calvino et al., 2011; White and Barton, 2011). The size exclusion limit (SEL) of PD allows the passive diffusion of small molecules ~1 kDa in size. This presents an impediment to virus movement because virus structures that are hypothesized to move between cells require a far larger SEL. The MP of TMV modifies the SEL of the PD to facilitate virus movement through PD and fluorescently labeled MP is observed in PD after virus spread has occurred (reviewed in Benitez-Alfonso et al., 2010; Niehl and Heinlein, 2011). The MP itself also can move between cells when ectopically expressed (reviewed in Niehl and Heinlein, 2011). The increase in PD SEL during TMV spread is transient, returning to a restricted size after passage of the infection front as measured by fluorescence expressed from the TMV-GFP genome (Oparka et al., 1997). Microfilament antagonists lead to a significant increase in the PD SEL while those that stabilize microfilaments prevent the MP from increasing the PD SEL (White et al., 1994; Ding et al., 1996; Su et al., 2010). The TMV MP exhibits microfilament severing activity (Su et al., 2010). These findings, in total, suggest a mechanism for virus spread where TMV MP opens PD through its microfilament severing activity, mimicking the phenotype induced with microfilament antagonists (**Figure 1**). Su et al. (2010) hypothesized that the severing activity of the TMV MP was limited to the PD area by analogy with findings from a mutant MP of *Cucumber mosaic virus* altered in its ability to target the PD. This mutant MP, which also has severing activity, fragments microfilaments in the cytoplasm (Su et al., 2010). Besides the MP, evidence for the VRC moving between cells has been published (Kawakami et al., 2004), but there have been no subsequent reports supporting this finding. The 126 kDa protein fused with GFP has not been reported to move between cells during ectopic expression, suggesting that VRC intercellular movement requires expression of additional viral proteins (likely the MP) or the presence of the vRNA.

In addition to innate actin severing activity by the TMV MP that may enlarge PD, the TMV MP interacts with ankyrin repeat-containing protein, ANK, at the PD and this association is correlated with an increase the PD SEL (Ueki et al., 2010). ANK has multiple activities, including binding to and delivering chloroplasts to their destination, supporting disease resistance against bacteria and virus challenge and participating in reactive oxygen scavenging, but it does not have callose degrading activity. Over-expressing ANK in transgenic plants resulted in more extensive MP-YFP and TMV-DsRed spread between cells. During transient expression of ANK and MP, the level of callose in the neck region of the PD decreased. Previously it was shown that enhanced TMV intercellular spread was correlated with enhanced expression of the callose-degrading enzyme, β -1, 3-glucanase (Bucher et al., 2001). Whether predominantly cytoplasmic ANK, through its MP interaction and the MP interaction with membrane, targets β -1, 3-glucanase in ER-derived vesicles to the cell wall or if ANK functions directly to inhibit callose synthase activity, remains to be determined (Ueki et al., 2010; **Figure 1**). Interactions of the TMV MP with other host proteins that influence TMV spread have been described (e.g., pectin methyl-esterase; Chen et al., 2000). However, in many instances cell biological studies to observe the interaction between the MP and host proteins within a live cell have not been conducted to further determine the location where the interaction may influence intercellular spread.

Regarding microtubules and TMV intercellular movement, there is evidence that MP interaction with the microtubule end-binding protein 1a (EB1a) is important for TMV spread. Brandner et al. (2008) determined that EB1a-GFP and MP-GFP form complexes both *in vitro* and *in vivo*. EB1a-GFP sub-cellular localization during TMV infection was altered from end labeling comet-like structures representing growing microtubules to labeling the length of microtubules. The length-wise decoration of microtubules was associated with a co-localization of MP-RFP. This unexpected re-localization of the EB1a protein at the infection front was correlated with an inhibition of virus intercellular spread. Ouko et al. (2010) determined that a mutant tobacco expressing a detyrosinated α-tubulin had a slower moving GFP-EB1 and inhibited intercellular spread by TMV. These studies suggest that modified microtubule dynamics inhibits TMV intercellular spread, perhaps by inhibiting microtubule polymerization that normally would push the VRC along the ER during intracellular transport (reviewed in Peña and Heinlein, 2012; **Figure 1**). These findings support those using a mutant strain of TMV whose temperature sensitive intercellular movement is correlated with the temperature sensitive localization of MP with microtubules (Boyko et al., 2007). However, other studies using pharmacological agents or silencing of the α-tubulin gene found no effect of these treatments on TMV intercellular spread or on the presence of MP in PD (Gillespie et al., 2002). Additionally, a virus expressing a modified MP with limited affinity for microtubules displayed enhanced intercellular spread (Gillespie et al., 2002). These and other findings from studies of a microtubule-binding protein, MPB2C, which binds to the MP and when overexpressed has a negative effect on intercellular movement of a related tobamovirus (Ruggenthaler et al., 2009), led to the suggestion that the function of microtubules in TMV spread is for degradation of the MP. Recently, it was determined that a CELL-DIVISION CYCLE protein48 (CDC48) from *Arabidopsis*, which localizes in the cytoplasm near the cortical ER network, interacts with the TMV MP to extract it from ER for its subsequent accumulation on microtubules (Niehl et al., 2012). These authors also determined that overexpression of CDC48 in infected tissue inhibited virus spread. Thus, it appears that removal of the MP from the ER at the infection front and its movement to the microtubules through CDC48 activity is directed toward processing and possibly, degrading, the MP (**Figure 1**).

Microfilaments have been shown to be important for TMV intercellular movement, but the interpretation of their involvement in this activity is evolving. Findings from early studies using a GFP-labeled virus showed that disruption of microfilaments, through pharmacological methods or by silencing actin, inhibited sustained (2 days and beyond post-inoculation) virus intercellular movement (Kawakami et al., 2004; Liu et al., 2005). This inhibition in sustained intercellular spread was not associated with a decrease in virus accumulation per cell that would affect virus movement or prevent VRC formation (Liu et al., 2005). In addition, the sustained virus intercellular movement was not correlated with VRC size (Liu et al., 2005). This movement required myosin motor activity and specifically myosin XI-2 motor activity (Kawakami et al., 2004; Harries et al., 2009). Surprisingly, intercellular movement of the related tobamovirus, TVCV, was unaffected by disruption of microfilaments or silencing of any myosin studied to date (Harries et al., 2009). During this time, it also was shown that TMV was not inhibited in spread early after LatB treatment (i.e., movement up to 24 h after treatment; Hofmann et al., 2009). In addition, these researchers determined that actin binding domain 2 (ABD2)-GFP, expressed in transgenic plants, inhibited TMV movement. They concluded that this disruption in virus movement was primarily due to a loss of membrane fluidity caused by the ABD2-GFP marker and that TMV intercellular movement was predominantly influenced by membrane diffusion characteristics. Thus, results from studies of both TVCV and early TMV intercellular movement suggest membranes as the predominant vehicle controlling tobamovirus intercellular movement. These findings also support those from a previous study showing that both the viral replication proteins and MP are necessary to allow maximum diffusion through PD of GFP-fused probes representing soluble ER membrane-bound proteins (Guenoune-Gelbart et al., 2008). It was concluded that the results best support a model in which the virus complex, perhaps consisting of viral RNA, MP and other proteins, diffuses on the ER membrane within the PD from infected to uninfected cells driven by a concentration gradient (Guenoune-Gelbart et al., 2008). The involvement of the ER directly in virus movement, without prior transport through Golgi, is supported by the finding that inhibition of the COP II transport system through expression of a dominant negative GTPase mutant protein, Sar1, did not inhibit sustained TMV intercellular movement (Genovés et al., 2010).

Irrespective of the mechanism of early movement between cells by TMV, an explanation of this virus’s requirement for microfilaments for sustained movement is required. Harries et al. (2010) suggested that the influence of microfilaments on this activity may be in preventing a stress response from occurring at the PD that later signaled to cells in advance of virus spread. These cells would then modify their metabolism in preparation for the arrival of the virus. Here we hypothesize that it is the transport of the VRC from the cell wall/PD area to an internal subcellular location on the actomyosin array that is necessary to prevent the stress signal from moving to the next cell (**Figure 1**). On treatment with LatB or silencing of myosin XI-2, the TMV VRCs would remain at the cell wall/PD interface, inhibiting normal cell–cell communications. This stress would be signaled to the cells in advance of the virus spread and modify these cells to inhibit subsequent virus spread. This hypothesis is supported by the finding that the VRCs of TMV move away from the cell wall as the infection passes (Szécsi et al., 1999). This interpretation would also accommodate the finding that early TMV movement is unaffected by actomyosin inhibitors since it would take some time to signal in advance of the infection front to stop virus movement. In addition, it would explain the lack of effect of LatB on TVCV movement since the 125 kDa protein, the homolog of the 126 kDa protein of TMV, does not form intracellular inclusions that associate with microfilaments (Harries et al., 2009) and the virus itself produces few visible VRCs (Resconich, 1961). The transport of the TMV VRC or its remnants from the wall may require membranes and vesicles since the TMV MP binds with a plant synaptotagmin *in vitro* (Lewis and Lazarowitz, 2010). Synaptogamins are a family of Ca^2^^+^- and lipid-binding proteins that modulate, through interaction with SNARE proteins, the fusion of vesicles with membranes (Chapman, 2008). A dominant negative synaptotagmin mutant caused depletion of endosomes and inhibited intercellular trafficking of the MP-GFP fusion (Lewis and Lazarowitz, 2010). The direct influence of synaptotagmin on virus physiology appears to be on the endocytic pathway, implying that the effect of the dominant negative synaptotagmin on virus movement is through blocking the recycling of membrane used by the virus to reach the cell wall/PD area, thus backing up the system (**Figure 1**). This hypothesis, suggesting a requirement for actomyosin-mediated vesicle trafficking of the VRC or VRC components from the wall membranes for sustained virus movement, can be evaluated through cell biological studies.

Regarding non-cytoskeletal or membrane-associated proteins and TMV intercellular movement, a gene encoding a class II KNOTTED1-like protein, NTH201, was cloned from *Nicotiana tabacum* and its expression levels were positively correlated with MP accumulation, VRC numbers and virus spread (Yoshii et al., 2008). NTH201 has no ability to traffic between cells or traffic GFP, unlike class I KNOX-like plant proteins, and the authors speculated that NTH201 may function as a transcription factor that helps to stabilize or fold MP and VRCs through its regulation of other genes. This mystery was partially explained when a second host factor, MPIP1, a DnaJ-like protein with potential chaperone activity, was determined to interact with TMV MP and NTH201 in a yeast-three hybrid screen (Shimizu et al., 2009). Silencing MPIP1 inhibits TMV spread, as determined by GFP fluorescence, and thus its silencing phenocopies observations of TMV spread when NTH201 expression was silenced. It is possible that an interaction between two host proteins and the TMV MP aid in transport of virus between cells (**Figure 1**).

## CONCLUSION

Cell biological studies over the last 20 years have tremendously aided our understanding of TMV accumulation and spread. Without advanced molecular and biochemical technologies allowing virus and virus component labeling and advanced imaging hardware our understanding of the individual processes during virus spread would be diminished. For example, if virus intercellular movement were studied by genetics alone the importance of the transport of TMV vRNA granules to the perinuclear region of the ER versus the transport of TMV VRCs and MPs to the PD could go unrecognized. This said, some cell biological studies have yielded conflicting or controversial results. This is especially true of pharmacological studies and researchers must carefully control as many variables in these studies as possible. In addition, conclusions from pharmacological studies should be verified using other methods. Use of novel virus labeling techniques and advanced microscopes will allow further advances in this area. For example, the identification of small fluorescent tags that do not influence the function of the viral protein to which it is fused will be helpful. The iLOV protein, derived from the blue light receptor phototropin and much smaller than GFP, has been available for some time and was a first step toward utilizing smaller fluorescent tags (Chapman et al., 2008). More recently a MYB-related transcription factor, Rosea 1, which is also smaller than GFP, has been placed in TMV as a marker for virus location (Bedoya et al., 2012). Advanced microscopes with super high resolution will allow us to more easily determine whether proteins are interacting or simply co-localizing (Tilsner and Oparka, 2010). Access to the *N. benthamiana* genome (Bombarely et al., 2012) will allow better identification of gene family members projected to influence virus transport and the ability to target individual members for knockdown, over-expression and labeling. Lastly, recent findings suggest that while transport of TMV to the PD is important, it is also important to understand what happens to the virus inclusions left in the cell after virus movement, since their proper degradation or storage may influence sustained intercellular movement by the virus. As an analogy, although humans can function well for a time in their home (i.e., cell) without working plumbing, a plugged drain, like a plugged PD, will eventually be noticed. With our improving technologies, resources, and knowledge the future is bright, literally, for cell biological studies on TMV accumulation and spread.

## Conflict of Interest Statement

The authors declare that the research was conducted in the absence of any commercial or financial relationships that could be construed as a potential conflict of interest.
